# Wafer‐Scale Replication of Plasmonic Nanostructures via Microbubbles for Nanophotonics

**DOI:** 10.1002/advs.202404870

**Published:** 2024-09-03

**Authors:** Jehwan Hwang, Yue Zhang, Bongjoong Kim, Jinheon Jeong, Jonghun Yi, Dong Rip Kim, Young L. Kim, Augustine Urbas, Gamini Ariyawansa, Baoxing Xu, Zahyun Ku, Chi Hwan Lee

**Affiliations:** ^1^ Weldon School of Biomedical Engineering Purdue University West Lafayette IN 47907 USA; ^2^ Optical Lens Materials Research Center Korea Photonics Technology Institute (KOPTI) Gwangju 61007 Republic of Korea; ^3^ Department of Mechanical and Aerospace Engineering University of Virginia Charlottesville VA 22904 USA; ^4^ Department of Mechanical and System Design Engineering Hongik University Seoul 04066 Republic of Korea; ^5^ School of Mechanical Engineering Hanyang University Seoul 04763 Republic of Korea; ^6^ Materials and Manufacturing Directorate Air Force Research Laboratory Wright‐Patterson Air Force Base Dayton OH 45433 USA; ^7^ Sensors Directorate Air Force Research Laboratory Wright‐Patterson Air Force Base Dayton OH 45433 USA; ^8^ Apex Microdevices 4871 Misrach CT West Chester OH 45069‐7755 USA; ^9^ School of Mechanical Engineering Purdue University West Lafayette IN 47907 USA; ^10^ School of Materials Engineering Purdue University West Lafayette IN 47907 USA; ^11^ Elmore Family School of Electrical and Computer Engineering Purdue University West Lafayette IN 47907 USA; ^12^ Birck Nanotechnology Center Purdue University West Lafayette IN 47907 USA

**Keywords:** metamaterials, microbubbles, nanophotonics, nano‐transfer printing, plasmonic nanostructures

## Abstract

Quasi‐3D plasmonic nanostructures are in high demand for their ability to manipulate and enhance light‐matter interactions at subwavelength scales, making them promising building blocks for diverse nanophotonic devices. Despite their potential, the integration of these nanostructures with optical sensors and imaging systems on a large scale poses challenges. Here, a robust technique for the rapid, scalable, and seamless replication of quasi‐3D plasmonic nanostructures is presented straight from their production wafers using a microbubble process. This approach not only simplifies the integration of quasi‐3D plasmonic nanostructures into a wide range of standard and custom optical imaging devices and sensors but also significantly enhances their imaging and sensing performance beyond the limits of conventional methods. This study encompasses experimental, computational, and theoretical investigations, and it fully elucidates the operational mechanism. Additionally, it explores a versatile set of options for outfitting nanophotonic devices with custom‐designed plasmonic nanostructures, thereby fulfilling specific operational criteria.

## Graphical abstract

A robust technique for the rapid, scalable, and seamless replication of quasi‐3D plasmonic nanostructures is presented straight from their production wafers using a microbubble process. The method enhances imaging and sensing performance in nanophotonic devices by simplifying integration, enabling defect‐free replication, and allowing substrate reuse, thereby advancing the capabilities and applications of nanophotonic technologies.

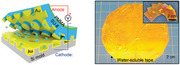

## 1 Introduction

Light manipulation plays a pivotal role in enabling the advancement of optical imaging and sensing across a wide range of nanophotonic devices, including biosensors, medical imaging equipment, optoelectronic sensors, and optical nanoantennas.^[^ ^1^, ^2^, ^3^, ^4^, ^5^, ^6^, ^7^, ^8^, ^9^ ^]^ These nanophotonic devices often rely on various plasmonic nanostructures such as nanopost arrays, nanohole arrays, nanoring arrays, and nanogratings that can enhance optical absorption, improve polarization sensitivity, and provide wavelength selectivity.^[^ ^10^, ^11^, ^12^, ^13^, ^14^, ^15^, ^16^ ^]^ Among those, quasi‐3D plasmonic nanostructures, when integrated with these optical devices, offer an innovative way to control light‐matter interactions at the nanoscale, surpassing the inherent optical properties of materials.^[^ ^17^, ^18^, ^19^, ^20^ ^]^ By combining upper and lower nanostructured arrays in repetitive quasi‐3D configurations, an additional resonance mode can be generated, leading to significant near‐field confinement and optical amplification effects at the resonance frequency.^[^ ^21^, ^22^, ^23^ ^]^ As a result, when carefully designed, these nanostructures can enhance optical performance and enable miniaturization, integration, and high‐speed operation beyond the constraints of traditional optical elements.

Existing polarization or hyperspectral LWIR sensors detect polarized light or specific wavelengths by mounting the optical system in front of the camera. This approach introduces several limitations, including increased cost, complexity, calibration requirements, and challenges in achieving real‐time detection. Our approach utilizes plasmonic films with nanostructures to enhance light absorption, improve polarization sensitivity, and provide wavelength selectivity. By monolithically integrating these plasmonic films into LWIR sensors, we significantly reduce the volume and weight of the sensors while adding various functionalities. This integration is made possible by advancements in nanotechniques, such as e‐beam lithography, laser direct writing, and nanoimprint lithography, which allow for the precise and large‐scale implementation of these complex nanostructures.^[^ ^24^, ^25^, ^26^ ^]^ However, existing nanopatterning methods face limitations when applied to various applications with specific requirements. E‐beam lithography, while renowned for its precision in creating small‐scale, complex patterns, becomes cost‐prohibitive and inefficient for large‐area applications, making it less practical for mass production. Additionally, its requirement for a conductive substrate for pattern fidelity poses a risk of damaging device circuits if a conductive coating is applied. On the other hand, nanoimprint lithography is better suited for mass production but encounters challenges with substrate scalability due to the direct pressure involved in the process, which can adversely impact device functionality, particularly in highly integrated systems. These limitations underscore the need for advanced lithography techniques that can achieve both scalability and functionality across a wide range of applications.

Quasi‐3D plasmonic nanostructures can be fabricated on either wafers or directly onto optical devices using advanced nanofabrication methods such as e‐beam lithography, laser direct writing, and nanoimprint lithography. However, the nanofabrication methods are intricate and time‐consuming, inevitably presenting scalability challenges. Moreover, these methods necessitate the use of corrosive chemicals and high temperatures during a series of etching, deposition, and patterning steps, which may cause damage to the intended optical devices. To circumvent these challenges, nano‐transfer printing methods, which utilize repetitive pick‐and‐place processes with an elastomeric stamp, have been used as a powerful method for stacking 2D plasmonic nanostructures into 3D configurations.^[^ ^27^, ^28^, ^29^, ^30^, ^31^, ^32^, ^33^, ^34^ ^]^ However, the heavy dependence on repeated stacking processes, which requires precise control of interfacial adhesions through physical or chemical treatments, as well as accurate nanoscale alignments of multiple layers onto a designated location, creates obstacles for widespread adoption.

Several alternative nano‐transfer printing methods have emerged to facilitate the physical separation of the entire quasi‐3D plasmonic nanostructures from their fabrication wafers.^[^ ^35^, ^36^, ^37^, ^38^ ^]^ A common approach involves the utilization of a chemically etchable sacrificial layer, enabling the subsequent detachment process.^[^ ^39^, ^40^ ^]^ The isolated nanostructures can then be integrated with the intended optical devices under mild conditions, such as in the air or underwater, effectively minimizing the risk of inducing physical, chemical, or thermal damage. Despite their applicability, these methods may prove inefficient for wafer‐scale production due to the slow chemical etching reaction and diffusion rates, leading to a lengthy peeling process that can span from multiple tens of minutes to several hours. This inefficiency stems from a combination of high costs, time constraints, and the complexities involved in implementing high‐throughput procedures. Therefore, there remains a need for the development of a rapid, scalable, and seamless transfer printing method that can seamlessly integrate quasi‐3D plasmonic nanostructures with diverse optical devices.

Here, we present a robust technique capable of replicating quasi‐3D plasmonic nanostructures at a 4‐inch wafer scale in less than 10 min directly from their production wafers using a microbubble process. The replicated nanostructures can then be seamlessly integrated with a wide variety of nanophotonic devices, including commercial infrared (IR) images and custom‐built polarimetric photodetectors, under ambient conditions. The working principle of this method hinges on employing microbubbles, which are byproducts of the water electrolysis occurring at the interface between the quasi‐3D plasmonic nanostructures and their fabrication wafers. This method offers several unique features: 1) rapid peeling from 4‐inch wafer‐scale Si molds, enabled by fast water electrolysis; 2) defect‐free peeling, with the maximum strain of the brittle material layer being less than 2%, which is lower than the fracture strain of the component brittle material (≈4%); 3) repetitive replication using reusable wafer molds, which reduces material waste and production costs; and 4) implementation of nanopattern structures and dimensions in various 3D configurations. As proof‐of‐concept demonstrations, we replicated a range of quasi‐3D plasmonic films from Si molds, each tailored for specific wavelength‐selective and polarization‐sensitive plasmonic functionalities. The replicated plasmonic films were then integrated with various optical devices, including 1) multimodal bandpass and polarization filters, 2) commercial IR thermal imaging cameras (A320 series; FLIR), and 3) custom‐built photodetectors such as long‐wavelength infrared type‐II superlattice (LWIR‐T2SL). The multimodal filters, upon integration, successfully demonstrated the ability to achieve simultaneous polarization sensitivity and bandpass filtering. Additionally, both the integrated IR thermal imaging camera and photodetector exhibited remarkable advancements in polarization performance, surpassing an extinction ratio of 1800. These results underscore its potential for widespread adoption in scalable, cost‐effective, and compact nanophotonic applications with enhanced functionality.

## 2 Results and Discussion

### 2.1 Wafer‐Scale Replication of Quasi‐3D Nanostructures via Microbubbles

**Figure 1:**
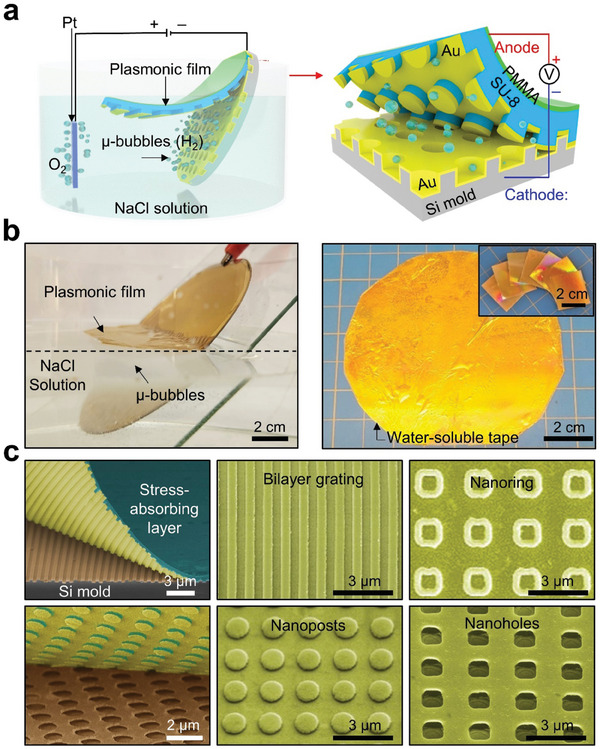
a) Schematic illustration of replicating a quasi‐3D plasmonic film from a 4‐inch wafer‐scale Si mold using the microbubble process. b) Photographs of a 4‐inch wafer‐scale quasi‐3D plasmonic film during the peeling process by microbubbles. c) SEM images of the transferred quasi‐3D plasmonic film showcase various nanostructure configurations, including four different patterns: bilayer gratings, nanorings, nanoposts, and nanoholes.

**Figure** **1**a schematically illustrates the process to replicate a wafer‐scale quasi‐3D nanostructure from a Si mold, leveraging microbubbles generated through the electrochemical decomposition of water (H_2_O) into hydrogen (H_2_) at the cathode. Initially, arrays of desired quasi‐3D plasmonic patterns were structured into the Si mold using standard nanolithography and dry etching techniques. Following this, thin layers of Ti/Au (5 nm/15 nm) and SiO_2_ (10 nm) were sputter‐coated onto the Si mold, serving as the anode electrode and a separation layer, respectively. The plasmonic films (e.g., Au film) were deposited using an e‐beam evaporator, and the IR optical materials (e.g., SU‐8) were spin‐coated to the specified thicknesses, completing the plasmonic film formation. A 1,000 nm‐thick layer of polymethyl methacrylate (PMMA) was subsequently spin‐coated on top as a stress‐absorbing layer, preparing the plasmonic film for detachment using microbubbles generated by water electrolysis. In the setup, a platinum (Pt) plate electrode and the Ti/Au electrode were used as the cathode and anode, respectively, with a 2 m NaCl aqueous solution acting as the electrolyte. A constant direct current (DC) voltage of 8 V was applied across the electrodes. The 8 V voltage was chosen for the actual production to enable efficient fabrication while ensuring the strain in the films below the failure strain. Under these conditions, the Ti/Au electrode was cathodically polarized, inducing the generation of microbubbles at the interface between the Ti/Au electrode and the SiO_2_ layer. This facilitated the separation of the entire plasmonic film from the Si mold. Representative scanning electron microscopy (SEM) images of each step are shown in Figure S1 (Supporting Information).

Figure 1b presents photographs of a representative plasmonic film, replicated from a 4‐inch‐wafer‐scale Si mold, utilizing microbubbles. A real‐time video capturing the replication process is shown in Movie S1 (Supporting Information). The plasmonic film was then affixed to a water‐soluble tape (Aquasol) for temporary handling to be cut into preferred dimensions for subsequent applications. Figure 1c provides the SEM images of the replicated plasmonic films featuring four different patterns, such as bilayer gratings, nanorings, nanoposts, and nanoholes. The nanopattern size was precisely controlled, with nanorings having a minimum line width of 330 nm and the diameters of nanoposts and nanoholes adjustable over a wide range of up to 1800 nm. Details of these nanostructures and their corresponding optical measurement and modeling results are shown in Figure S2 (Supporting Information). These findings indicate that the physical and optical characteristics of the replicated plasmonic films were well preserved, which proves a defect‐free replication process. Additionally, the Si master molds remained intact and defect‐free, thus enabling recycling for additional replication processes (Figure S3, Supporting Information). This recyclability presents an opportunity for reducing material waste and lowering production costs.

### 2.2 Theoretical and Computational Mechanism Analysis

The water electrolysis begins when the applied electrical voltage reaches a level that can provide the minimum energy needed to disrupt the thermodynamic equilibrium. This process generates H_2_ and oxygen (O_2_) gas microbubbles at the cathode and anode electrodes, respectively. The resulting microbubbles exert a consistent force that disrupts the adhesive bond between the plasmonic film and its Si mold, leading to interfacial delamination. With post‐nucleation, these microbubbles continue to grow until they detach from the interface. The resultant interfacial delamination distance during this time is represented as:

(1) $$L_{d}=r_{d}\;-r_{0}$$

where *r_d_* and *r*_0_ denote the detachment and nucleation radius of the microbubbles, respectively.^[^ ^41^ ^]^ The average interfacial delamination speed can be calculated as:

(2) $$\bar{v}=\frac{L_{d}}{T_{d}}\;$$

where *T_d_* is the total microbubble growth time before detachment. This overall growth time of microbubbles can be determined by:

(3) $$\int_{0}^{T_{d}}v_{g}dt=r_{d}\;-r_{0}$$

where *v_g_* is the microbubble growth rate. The net gas diffusion rate into the bubble can be determined by:

(4) $$J_{d}=J_{p}\;-\frac{V_{s}dc_{s}}{dt}$$

where the overall gas production rate is $J_{p}=\frac{I}{nF_{a}N_{b}}$, *I* is the current in the electrochemical cell, *N_b_* is the number of bubbles, *n* is the number of electrons involved in the reaction, and *F_a_* is the Faraday constant; *c_s_* is the gas concentration in the electrolyte and *V_s_* is the electrolyte volume. The gas diffused into the bubble will lead to the bubble growth and there is:

(5) $$J_{d}=\;4\pi r^{2}c_{b}v_{g}$$

where *r* is the bubble radius and *c_b_* is the gas concentration in the bubble. Therefore, the bubble growth rate becomes:

(6) $$v_{g}=\frac{I}{4\pi r^{2}c_{b}nF_{a}N_{b}}\;-\frac{V_{s}dc_{s}}{4\pi r^{2}c_{b}dt}$$

which gives the quantitative relation between *v_g_* and *I*.

**Figure 2:**
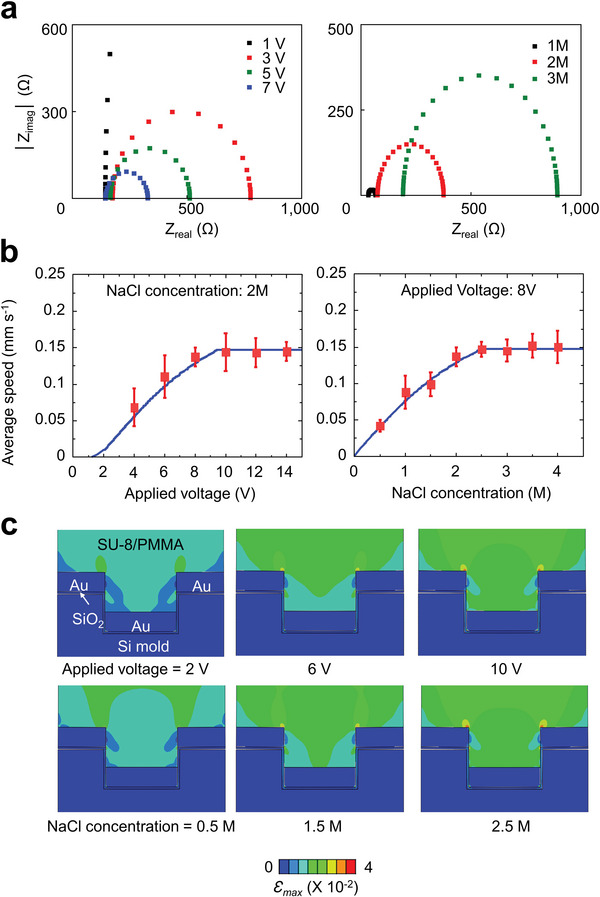
a) Impedance spectra obtained at different voltages and concentrations. b) Theoretical calculations and experimental measurements of the average interfacial delamination speed as a function of applied voltage and electrolyte concentration. Note here that the sample size was 2 × 2 cm and the samples were tested five times under each individual condition. c) Finite element analysis (FEA) results of strain distributions in the quasi‐3D plasmonic nanostructures film during the interfacial delamination under various applied voltages and electrolyte concentration conditions.

**Figure** **2**a illustrates the relationship between the current and the applied voltage, as well as the effect of varying conductive electrolyte concentrations. Figure 2b, derived from the preceding theoretical calculations, presents the average speed of interfacial delamination. The results indicate that the speed of interfacial delamination increases with an escalation in the applied voltage, only after reaching a critical electrical voltage required to disrupt thermodynamic equilibrium. However, after an initial surge, this speed plateaus, reaching a maximum value. Similarly, an increase in the NaCl concentration also elevates the average speed of interfacial delamination up to a certain limit, after which it plateaus. With further increases in the applied voltage and NaCl concentration, the current density in the electrochemical cell eventually hits a limiting threshold.^[^ ^41^ ^]^ This, in turn, facilitates peak rates of microbubble generation and growth, culminating in the maximum rate of interfacial delamination.

Figure 2c depicts the results of finite element analysis (FEA), displaying the strain distributions within the plasmonic film throughout the replication process at various applied voltages (2–10 V) and NaCl concentrations (0.5–2.5 m). Particularly, the maximum strain of the most brittle material component, SiO_2_, in the plasmonic film is less than 2%, which is far lower than its fracture strain under bending (≈4%).^[^ ^42^ ^]^ This low strain value indicates that the delamination process can be defect‐free. A real‐time video capturing these FEA results is shown in Movie S2 (Supporting Information). The results suggest that as the applied voltage and NaCl concentration increase, so does the strain. This can be attributed to the rising number of microbubbles and the force they apply in causing the interface to delaminate. Furthermore, Figure S4 (Supporting Information) presents the strain distribution in the plasmonic films with varied pitches.

### 2.3 Multimodal Quasi‐3D Metal‐Dielectric Nanoarrays

**Figure 3:**
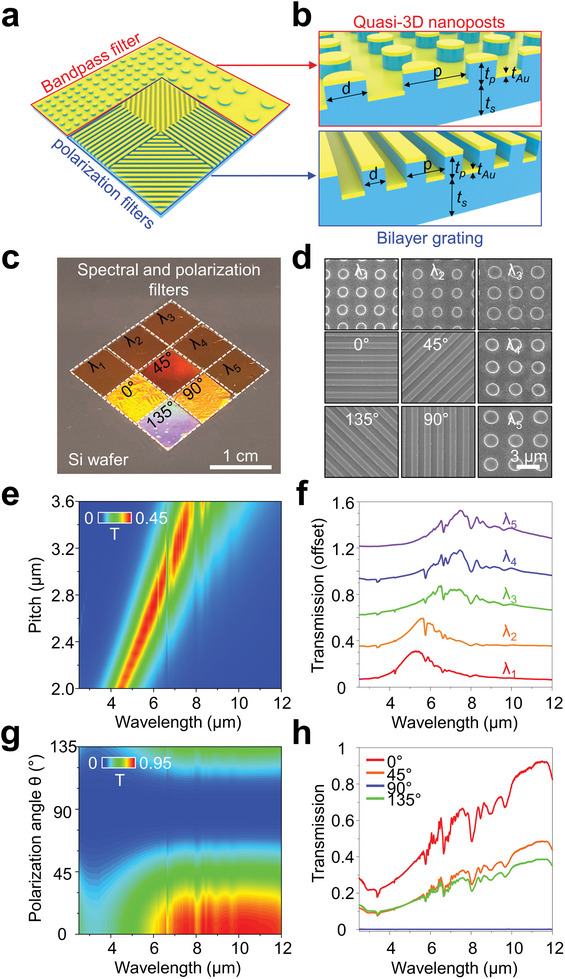
a) Schematic illustration of the spectral and polarization filters based on quasi‐3D plasmonic nanostructures, providing selection of polarization or wavelength. b) Enlarged schematic illustrations of the quasi‐3D nanoposts and bilayer gratings with periodicity (*p*), width (*d*), thickness of the posts (*t*_p_), spacing (*t*_s_), and gold layer thickness (*t*_Au_) representing respective dimensions. c) Photograph and d) SEM images of the fabricated spectral and polarization filters. e) Simulated and f) measured transmission spectra of the quasi‐3D nanoposts as a function of the period from 2.4 to 3.2 µm. g) Simulated and h) measured transmission spectra of the bilayer gratings as a function of the polarization angle.

A distinctive advantage of our approach is that it allows for the separate preparation of varied plasmonic patterns within quasi‐3D nanostructures which can then be assembled in a targeted manner akin to cut‐and‐paste. This capability is particularly beneficial in constructing complex optical systems for high‐precision biosensing, sophisticated medical imaging, and telecommunications, where tailored manipulation of light is essential. The availability of an array of spectral and polarization filters grants precise control over light interactions, critical for applications that demand heightened selectivity and sensitivity, thereby facilitating the isolation of specific wavelengths or polarizations to enhance device resolution and functionality. To increase polarization sensitivity, we selected a bilayer grating structure with metal lattices arranged along a single axis. Additionally, we chose a quasi‐3D nanopost that allows control of the resonance frequency by varying the period of the nanostructure for wavelength selectivity. **Figure** **3**a presents a demonstration illustrating an assortment of spectral and polarization filters based on quasi‐3D plasmonic nanostructures, grouped into two categories: bandpass and polarization filters. The polarization filters are composed of a 2 × 2 array of four bilayer grating elements, each set to a different orientation (0°, 45°, 90°, and 135°) to provide specific linear polarization data. Adjacent to this array lies a series of five bandpass filters, each featuring a unique nanopost array structure. Figure 3b delineates the structural parameters of the quasi‐3D plasmonic nanostructures, such as the periodicity (*p*), gold diameter (*d*), post thickness (*t*_post_), gold thickness (*t*_Au_), and spacer thickness (*t*_s_).

Figures 3c,d showcase digital and top‐view SEM imagery of the bandpass and polarization filters, highlighting the bilayer gratings and nanoposts. For each geometric configuration of bilayer gratings (polarizer orientation at 0°, 45°, 90°, and 135°) and nanoposts (*p* ranging from 2.4 to 3.2 µm), a plasmon film segment was excised and transposed onto a Si wafer, with specific parameters for bilayer gratings at *p* = 850 nm, *w* = 425 nm, *r* = 0.5, *t*_Au_ = 100 nm, and *t*_post_ = 200 nm. Figures 3e,f reveal both simulated and actual transmission spectra, derived from varying the pitches of chosen post arrays. To adjust both the peak wavelength and the constant transmission of the peak wavelength, the periodicity of the nanoposts was adjusted while keeping other geometric parameters (i.e., spacer thickness and width) fixed (Figure S5, Supporting Information). Selected plasmon filters with pitches from 2.4 to 3.2 µm display a near‐linear redshift in peak transmission from 4.9 to 6.8 µm as the period extends from 2 to 4 µm, with the peak resonance predominantly influenced by the post array period. Figures 3g,h present simulated and measured transmission spectra, adjusted for the polarization angle of the bilayer gratings. The documented TM transmission (I_0°_) and extinction ratio (I_0°_/I_90°_) were 25% (50%) and 643 (855) for wavelengths of 4 µm (8 µm), respectively. Thus, multimodal filters consisting of the bilayer gratings and nanoposts materialize as polarization‐sensitive bandpass filters, furnishing a dual response of polarization sensitivity and wavelength selectivity.

### 2.4 Multimodal Design of Polarization‐Sensitive Bandpass Filters

**Figure 4:**
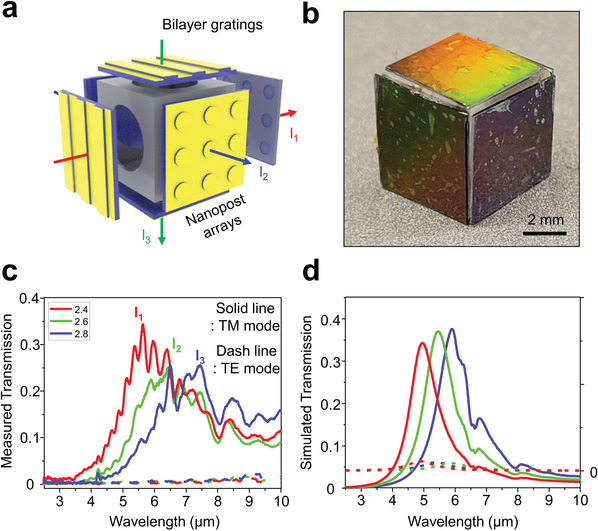
a) Schematic representation of a polarization‐sensitive bandpass filter composed of the quasi‐3D bilayer gratings and nanoposts modules. Strategically assembled filters demonstrate various filtering effects through the combination of modules. b) Photograph of a polarization‐sensitive bandpass filter with the quasi‐3D filters attached to a cube (5 × 5 × 5 mm). c) Simulated and d) experimentally measured transmission of the polarization‐sensitive bandpass filter as a function of wavelength for transverse magnetic (TM) and transverse electric (TE) polarized light.

Polarization‐sensitive bandpass filters with a multimodal configuration are critical components in various optical systems.^[^ ^2^, ^19^, ^43^ ^]^ They enhance imaging capabilities, improve communication technologies by enabling wavelength division multiplexing, and facilitate advanced sensors in scientific research and pragmatic applications ranging from medical diagnostics to environmental monitoring.^[^ ^1^, ^3^, ^4^ ^]^ **Figure** **4**a illustrates the schematic of such a polarization‐sensitive bandpass filter, demonstrating a multimodal design of quasi‐3D bilayer gratings and nanoposts modules. The quasi‐3D filters, meticulously replicated from their own Si molds, were accurately positioned and affixed to the surfaces of a hollow cube (5 × 5 × 5 mm^3^). This strategic assembly situates a wavelength selectivity filter on one cube face while a polarization sensitivity filter is placed on the opposite face, as shown in Figure 4b. Figures 4c,d detail the simulated and experimentally measured transmission spectra for transverse magnetic (TM) and transverse electric (TE) polarized light, respectively, as they interact with the structure. The quasi‐3D nanoposts and bilayer gratings are precisely engineered with dimensions: (i) periodicity (*p*) of 1.8 µm, width (*w*) as half of the periodicity, thickness of the posts (*t*_p_) at 200 nm, spacing (*t*_s_) at 500 nm, and a gold layer (*t*_Au_) of 50 nm, (ii) periodicity (*p*) of 850 nm, width (*w*) as half of the periodicity, thickness of the posts (*t*_p_) at 200 nm, and a gold layer (*t*_Au_) of 100 nm. The results highlight a bandpass filtering effect in the IR range with a peak wavelength at 5 µm for the quasi‐3D nanoposts, alongside a full width at half maximum (FWHM) at 1.6 µm. The transmission for TE‐polarized light at this peak wavelength is a mere 0.017%, with an extinction ratio of 1200. This distinct feature of our approach presents exceptional control over the polarization and wavelength of the transmitted light, which is integral to the development of highly selective and efficient optical filters. In addition, our approach demonstrates the feasibility of transferring the nanostructures onto specific receiving substrates, such as cubes or various optical modules.

### 2.5 Enhancement of LWIR Microbolometer Performance

**Figure 5:**
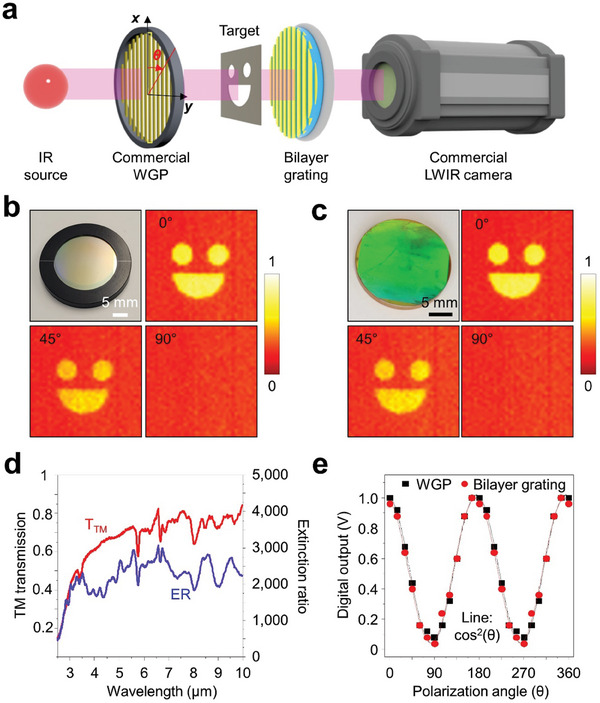
a) Schematic representation of an experimental setup to characterize the linear polarization performance of the transferred plasmonic film, featuring bilayer gratings, mounted on a zinc selenide window with an anti‐reflective coating. The captured IR images corresponding to four polarization directions (*θ* = 0°, 45°, 90°, and 135°) interacted with b) a reference commercial wire grid polarizer and c) the fabricated bilayer grating. d) Measured TM transmission and extinction ratio for the bilayer gratings. e) Polarization characteristics in the data extracted from the IR images of the fabricated bilayer grating with the angle of linearly polarized light.

The simple cut‐and‐pate transfer process of our approach simplifies the integration of quasi‐3D plasmonic filters into a variety of optical imaging devices and sensors, markedly enhancing their imaging and sensing performance beyond the capabilities of traditional methods. Such plasmonic filters are especially vital in applications involving long‐wavelength infrared (LWIR) microbolometer cameras, where they confer selective polarization control and augmented spectral response, resulting in superior thermal imaging resolution and heightened detection sensitivity. **Figure** **5**a illustrates an experimental setup facilitating the component‐level validation of the transferred plasmonic film, featured with bilayer gratings, onto a zinc selenide (ZnSe) window with an anti‐reflective coating, to appraise their optical performance. Commencing with unpolarized IR radiation from a hotplate set at 40 °C, the radiation traverses a commercial wire grid polarizer to yield linearly polarized IR radiation. This radiation's polarization orientation is fine‐tuned by adjusting the angle between the wire grid polarizer's axis and the engineered bilayer grating. Captured by an IR camera system are the images of the polarized radiation post interaction with the reference commercial wire grid polarizer (Figure 5b). Figure 5c shows the IR images for polarization angles of 0°, 45°, 90°, and 135° of the fabricated bilayer grating, while Figure 5d presents the measured TM transmission and extinction ratio for the bilayer gratings endowed with the 200 nm spacer—demonstrating a TM transmission exceeding 50% and an extinction ratio greater than 1000 within the LWIR spectrum. Figure 5e shows the data extracted from the IR image of the fabricated bilayer grating, which displays polarization characteristics that follow the Malus' Law for linear polarization angle. These demonstrations underscore a definitive advantage of our approach: a marked improvement in LWIR camera functionality, potentially establishing a new standard for thermal imaging excellence.

### 2.6 Enhanced LWIR Detection with Monolithically Integrated Photodetector

**Figure 6:**
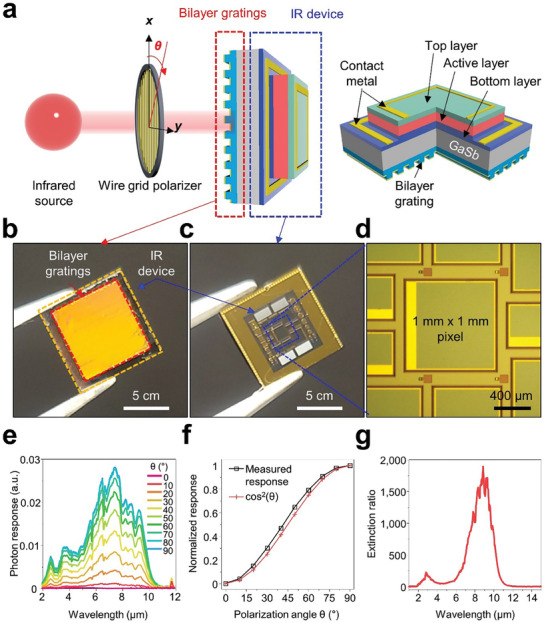
a) Schematic representation of an experimental setup with a monolithically integrated, polarization‐sensitive LWIR‐T2SL (Long‐Wavelength Infrared Type‐II Superlattice) photodetector. b) Photograph of the front and c) back of the monolithically integrated, polarization‐sensitive LWIR‐T2SL photodetector. d) Optical microscope image of a unit pixel among various mesas of the monolithically integrated, polarization‐sensitive LWIR‐T2SL photodetector. e) Measured photon response spectrum, f) photon response at specific wavelengths, and g) extinction ratio of the monolithically integrated, polarization‐sensitive LWIR‐T2SL photodetector.

To affirm the device‐level efficacy of our approach, we integrated a plasmonic film, featured with bilayer gratings, into a custom‐designed long‐wavelength infrared (LWIR) InAs/GaSb type‐II superlattice (T2SL) based single pixel device (SPD). The schematic depiction of our experimental arrangement is presented in **Figure** **6**a, which illustrates the layout of the LWIR‐T2SL SPD, with its intricate layers highlighted on the right. The InAs/GaSb T2SL structure, developed on a GaSb substrate, comprises a bottom contact layer, an absorption layer, and a top contact layer, all precisely engineered via molecular beam epitaxy (MBE). Sizes of the LWIR‐T2SL‐based SPDs range from 500 × 500 to 1000 × 1000 µm^2^, and their fabrication process is elaborated upon in Figure S6 (Supporting Information). Figures 6b–d exhibits the front and back views of the monolithically integrated, polarization‐sensitive LWIR‐T2SL photodetector. Figure 6e displays the photodetector's photon response measurements at various polarization angles from 0° to 90° at 77 K, demonstrating adherence to the Malus’ law (Figure 6f).^[^ ^42^ ^]^ The fabricated polarization‐sensitive LWIR‐T2SL photodetector exhibits high polarization sensitivity regardless of mesa size (Figure S7, Supporting Information), indicating potential applications in high‐resolution image sensors. A notable achievement of this integration is the attainment of an impressive extinction ratio of ≈1800 at the specific wavelength of 8.2 µm (Figure 6g), underscoring the superior performance of the polarization‐sensitive LWIR‐T2SL SPD. Table S1 (Supporting Information) presents a comparative study of the extinction ratio between the sensor presented in this study and previous works for polarimetric IR imaging applications. The sensor reported here exhibits substantially higher polarization sensitivity and an enhanced extinction ratio compared to previous references. Notably, it achieves this without adding general semiconductor processes to highly integrated devices, and it also enables the creation of integrated nanophotonics without generating defects. Instead of repeating the semiconductor process for nanopatterning on the LWIR‐T2SL photodetector, various plasmonic patterns can be prepared separately and assembled efficiently as intended, like cut‐and‐paste. This exceptionally high extinction ratio validates the efficacy of our approach and reinforces its potential for significant advancements in infrared detection and imaging technologies.

## 3 Conclusion

We present a robust, rapid, and scalable technique that seamlessly integrates quasi‐3D plasmonic nanoarrays onto a variety of substrates. Our approach leverages the microbubble‐induced interfacial delamination process in an electrolyte solution and circumvents the need for harsh chemicals and high‐temperature processing, resulting in defect‐free integration and the ability to reuse donor substrates, hence demonstrating both reliability and environmental friendliness. A thorough body of evidence, comprising experimental validations, computational simulations, and theoretical analyses, is presented to shed light on the operational principles, optimize conditions, and understand the intricate design trade‐offs involved. The adaptability and scalability of this process make it a formidable tool for the realization of complex 3D nanoarchitecture and the advancement of nanophotonics. By enabling rapid and efficient replication of quasi‐3D plasmonic films, we have demonstrated enhanced functionalities in a range of nanophotonic devices that previously faced significant integration challenges. The improvements observed in polarization performance and the achievement of high extinction ratios in IR imaging and photodetection exemplify the practical applications of this technology. Our findings not only pave the way for new research avenues in the field of nanophotonics but also contribute to the evolution of current and future technologies, offering significant implications for the development of compact, cost‐effective, and high‐performing optical devices.

## 4 Experimental Section

### Fabrication of Donor Si Substrates

Thin films of Ti/Au (5/15 nm thick) and SiO_2_ (10 nm thick) were deposited on a Si master mold on a 4‐inch Si wafer using a sputter to serve as an anode electrode and separation layer, respectively. A plasmonic Au film (50 nm thick) was deposited using an e‐beam evaporator at a deposition rate of 0.3 Å/s. SU‐8 (≈500 nm thick) and PMMA (50 nm thick) were spin‐coated to serve as infrared (IR) transparent dielectric spacers and stress‐absorbing layers, respectively. The entire structure was immersed in a solution of sodium chloride (NaCl) electrolyte solution with positive and negative potentials (4–6 V) applied to an electrolyte solution and anode electrode, respectively. The following electrochemical reactions occurred.

(7) $$2H_{2}O\;-\;4\;e^{-}=O_{2}+4H^{+}\left(\mathrm{Anode}\right)$$

(8) $$4H_{2}O+4e^{-}=2H_{2}\left(\mathrm{Microbubbles}\right)+4{\mathrm{OH}}^{-}\left(\mathrm{Cathode}\right)$$

The resulting microbubbles (H_2_) allowed the plasmonic film to be physically separated from the Si master mold without a defect. The plasmonic bilayer structure was then transferred onto a receiver substrate of interest.

### Computational Analysis

The FEA was performed by using the ABAQUS/standard package to simulate the interfacial delamination between films and substrates. In FEA simulations, the mechanical modulus (*E*) and the Poisson's ratio (*ν*) were *E* = 3.6 GPa and *ν* = 0.22 for SU‐8 film, *E* = 3.0 GPa and *ν* = 0.35 for PMMA film, *E* = 79 GPa and *ν* = 0.42 for Au film, and *E* = 75 GPa and *ν* = 0.17 for SiO_2_ film.^[^ ^42^, ^44^, ^45^ ^]^ The electrode substrates were considered as a rigid material. The films were meshed with four‐node bi‐linear plane strain elements. At least four layers of elements were used along the thickness in the thin film to well capture the through‐thickness stress distribution and bending deformation and mesh convergence was studied to confirm the sufficiency of discretization of the model. The interfacial interaction between the plasmonic film and the electrode substrate was described using the cohesive zone model (CZM). In the cohesive zone model, the interfacial traction‐separation relation was characterized by the following two key parameters: 1) cohesive strength (*σ*_0_, the maximum traction in the traction‐separation curve), and 2) critical energy release rate (*Γ*_c_, the area of the traction−separation curve), which were coupled with the electrochemical reaction.^[^ ^42^ ^]^ When the interface energy release rate reaches the critical energy release rate *Γ*_c_, the interfacial traction drops to zero, leading to a complete separation. To delaminate the interface in the FEA model, displacement loading was applied on the plasmonic film while the donor Si substrate was fixed.

### Numerical Simulation of Transmission

The optical properties of plasmonic nanostructures were verified through a numerical calculation carried out by the finite element method, utilizing the commercial 3D electromagnetic analysis software package (CST Studio Suite, Simulia). A frequency domain solver was employed to solve the model, and the structural parameters were based on the experimental results. The perfect electric conductor (PEC) boundary condition (*E*_t_ = 0) was applied for the *xz*‐plane, and perfect magnetic conductor (PMC) boundary conditions (*H*_t_ = 0) were applied for the *yz*‐plane. The dielectric functions of bulk gold in the infrared region were described according to the Drude model, with a plasma frequency of *w*_p_ = 1.38 × 10^16^ Hz and a collision frequency of *w*_c_ = 5.71 × 10^13^ Hz.^[^ ^46^ ^]^ The silicon substrate and spacer layer had frequency‐independent refractive indices of 3.4 and 1.54, respectively. The refractive index of the background material was set to 1. Subsequently, transmission data were acquired.

### FTIR Measurements

Transmission spectra were characterized with a Nicolet 6700 Fourier transform infrared (FTIR) spectrometer. The spectral range was 2.5–10 µm wavelength, and the resolution was set at 4 cm^−1^ (256 scans). For the normal incidence of the linearly polarized light source, a commercial wire grid polarizer was mounted in front of the source, configured at 90° for TE polarized light and 0° for TM polarized light. The polarization characteristics of the sample were determined by varying the angle of the manufactured sample between 0 and 90°.

### Fabrication of MWIR‐T2SL‐Based HPD

The material for the MWIR‐T2SL‐based HPD, consisting of bottom contact, absorption, and top contact layers, was grown on an *n*‐type GaSb wafer using molecular beam epitaxy (MBE). The device mesa was defined using conventional photolithography and phosphoric acid‐based wet chemical etching. Ohmic contacts were then formed on the top and bottom contact layers through the deposition of Ti/Pt/Au metal using an e‐beam evaporator.

### Characterization of MWIR‐T2SL‐Based HPD

To characterize the detectors, the photocurrent was measured using the Nicolet 6700 model Fourier transform infrared spectroscopy (FTIR) spectrometer. To electro‐optically characterize the fabricated T2SL pixeled detector, the Nicolet 6700 FTIR Spectrometer, equipped with a current amplifier (Keithley Model 428), was employed. Following the loading of the device into the cryostat, the vacuum is maintained, and subsequently, the temperature of the device is lowered to 77 K using liquid nitrogen to study the spectral response of T2SL with an applied bias of 0 mV.

### Statistical Analysis

Data processing and statistical analysis were conducted using OriginPro software. The measured data were expressed as mean ± standard deviation (SD) following analysis.

## Conflict of Interest

The authors declare no conflict of interest.

## Author Contributions

J.H., Y.Z., and B.K. contributed equally to this work. B.X., Z.K., and C.H.L. conceptualized the project. J.H., Y.Z., B.K., B.X., Z.K., and C.H.L. worked on the design, fabrication, and characterization of the plasmonic device. J.J. and J.Y. prepared samples and performed optical measurements. J.H. and Y.Z. conducted analytical modeling. G.A. fabricated and characterized the IR devices. D.R.K., Y.L.K., and A.U. analyzed the data and wrote the manuscript. All authors contributed to writing the manuscript. B.X., Z.K., and C.H.L. supervised the research and participated in writing the manuscript. All authors reviewed and commented on the manuscript.

## Supporting information

Supporting Information

Supplemental Movie 1

Supplemental Movie 2

## Data Availability

The data that support the findings of this study are available in the supplementary material of this article.
