# Supplementary material for: Wafer‐Scale Replication of Plasmonic Nanostructures via Microbubbles for Nanophotonics
Source: Adv Sci (Weinh). 2024 Sep 3;11(40):2404870. doi: 10.1002/advs.202404870 (PMC11516140; doi:10.1002/advs.202404870)
Supplement: Supplementary file 1 — Supporting Information [file ADVS-11-2404870-s002.docx]

Supporting Information

Wafer-scale replication of plasmonic nanostructures via microbubbles for nanophotonics

Jehwan Hwang^1,2‡^, Yue Zhang^3‡^, Bongjoong Kim^1,4‡^, Jinheon Jeong^1^, Jonghun Yi^5^, Dong Rip Kim^5^, Young L. Kim^1^, Augustine Urbas^6^, Gamini Ariyawansa^7^, Baoxing Xu^3*^, Zahyun Ku^8*^, Chi Hwan Lee^1,9,10,11*^

^1^Weldon School of Biomedical Engineering, Purdue University, West Lafayette, IN, 47907, USA.

^2^Optical Lens Materials Research Center, Korea Photonics Technology Institute (KOPTI), Gwangju 61007, Republic of Korea,

^3^Department of Mechanical and Aerospace Engineering, University of Virginia, Charlottesville, VA, 22904 USA.

^4^Department of Mechanical and System Design Engineering, Hongik University, Seoul, 04066, Republic of Korea.

^5^School of Mechanical Engineering, Hanyang University, Seoul 04763, Republic of Korea.

^6^Materials and Manufacturing Directorate, Air Force Research Laboratory, Wright-Patterson Air Force Base, Dayton, OH, 45433, USA.

^7^Sensors Directorate, Air Force Research Laboratory, Wright-Patterson Air Force Base, Dayton, OH, 45433, USA.

^8^Apex Microdevices, West Chester, OH, 45069, USA.

^9^School of Mechanical Engineering, Purdue University, West Lafayette, IN, 47907, USA.

^10^School of Materials Engineering, Purdue University, West Lafayette, IN, 47907, USA.

^11^Birck Nanotechnology Center, Purdue University, West Lafayette, IN, 47907, USA.
E-mail: zahyun@apexmds.com (Z.K.); bx4c@virginia.edu (B.X.); lee2270@purdue.edu (C.H.L.)

**
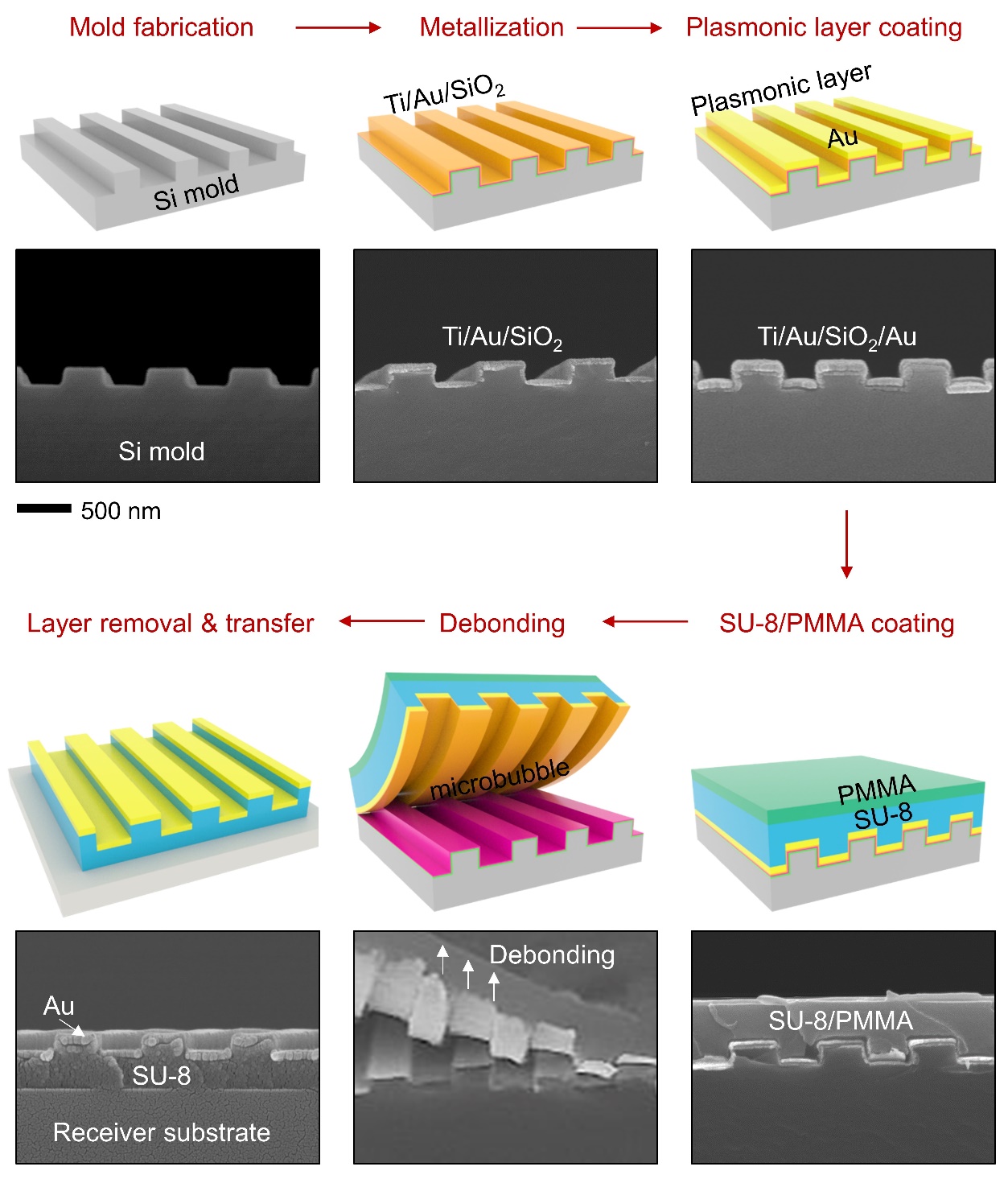
**

**Figure S1.** Schematic illustrations and SEM images describing the wafer-scale replication of quasi-3D nanostructures via the microbubble process.


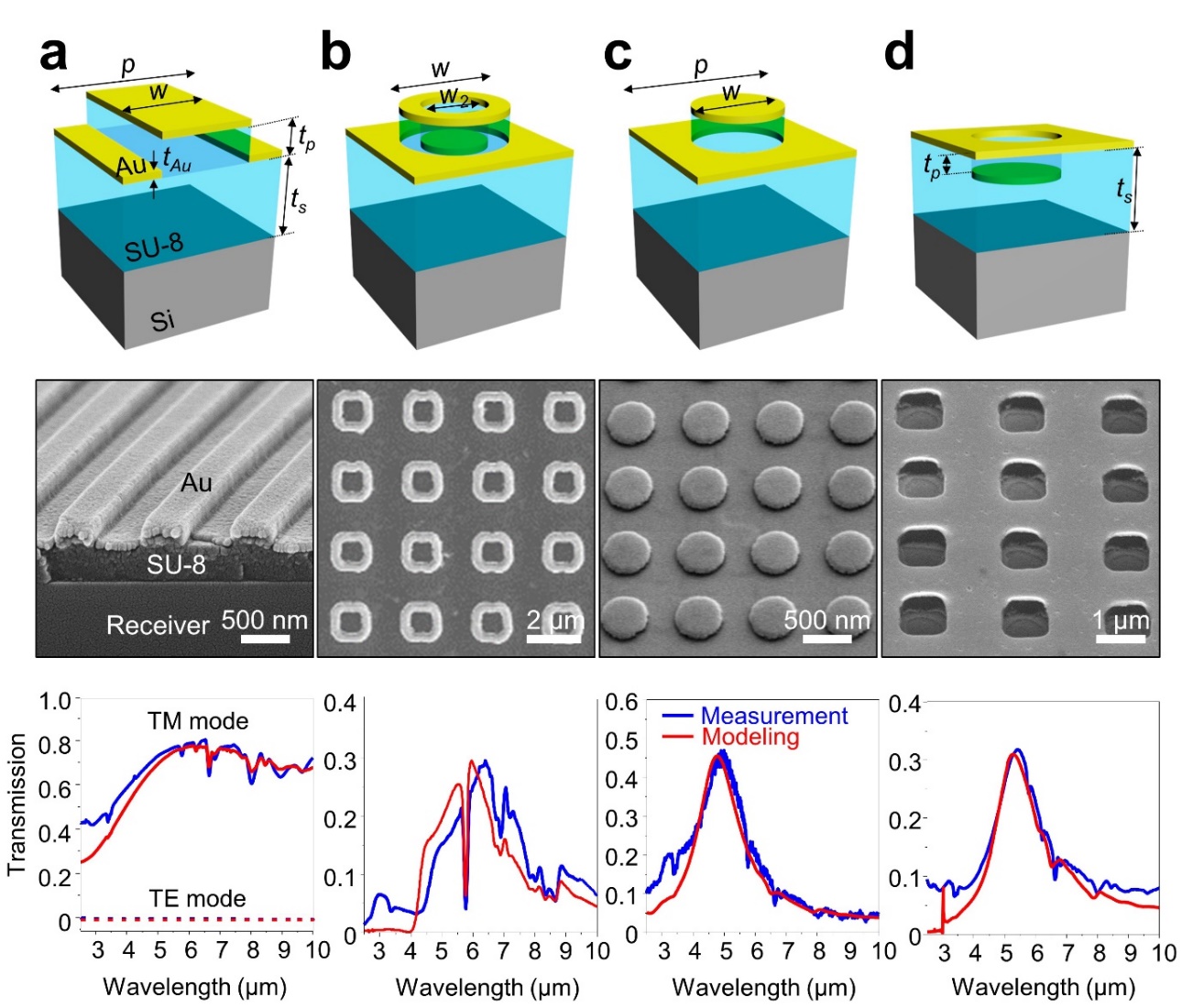


**Figure S2**. Schematic illustrations, SEM images, and measured transmissions of the unit cells of customized plasmonic films transferred onto various nanostructures, including (a) bilayer gratings, (b) nanorings, (c) nanoposts, and (d) nanoholes.


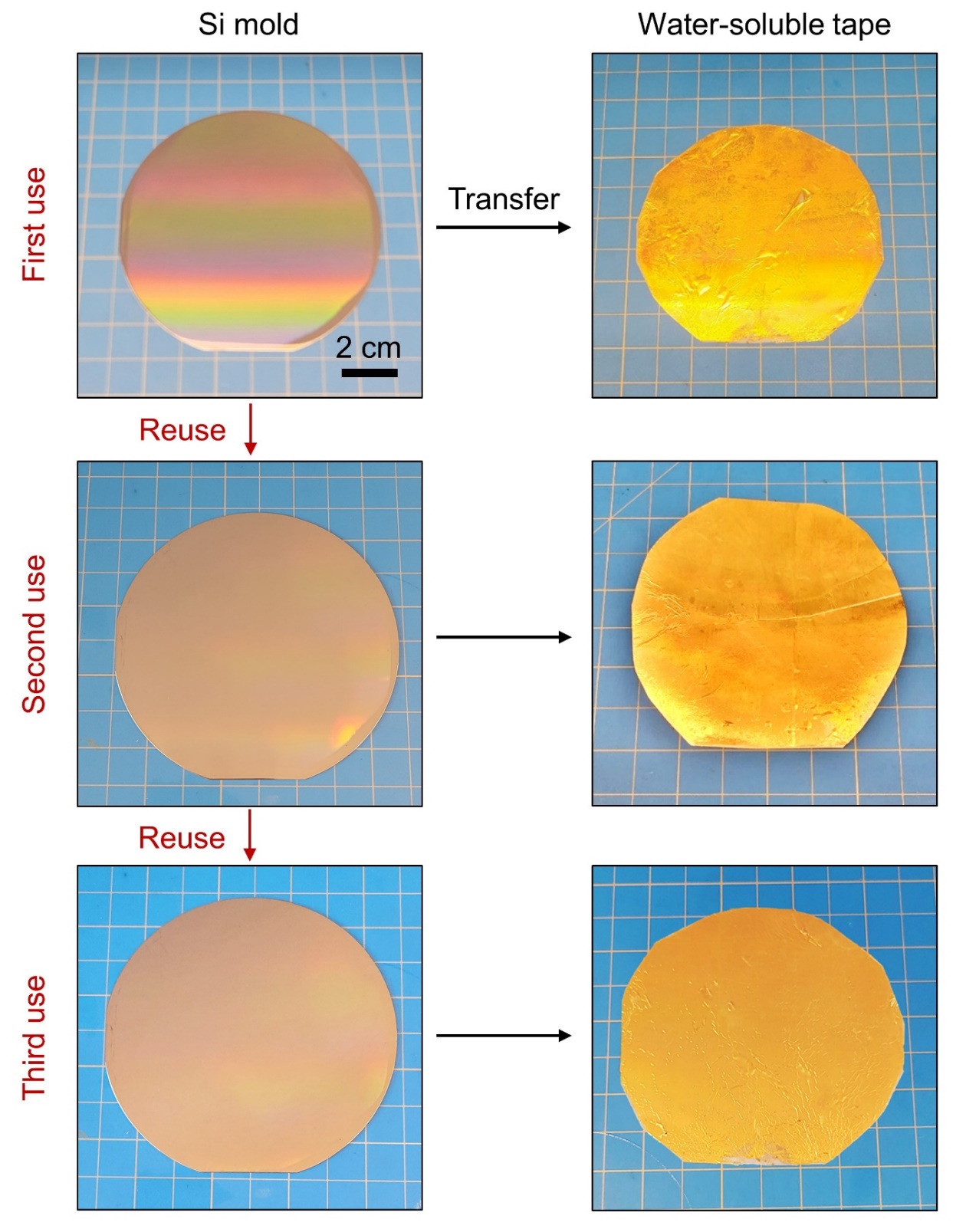


**Figure S3.** Photographs of the transferred quasi-3D plasmonic filters and 4-inch wafer-scale Si molds after the repeated transfer of the quasi-3D plasmonic film onto receiver double side polished (DSP) silicon wafers.


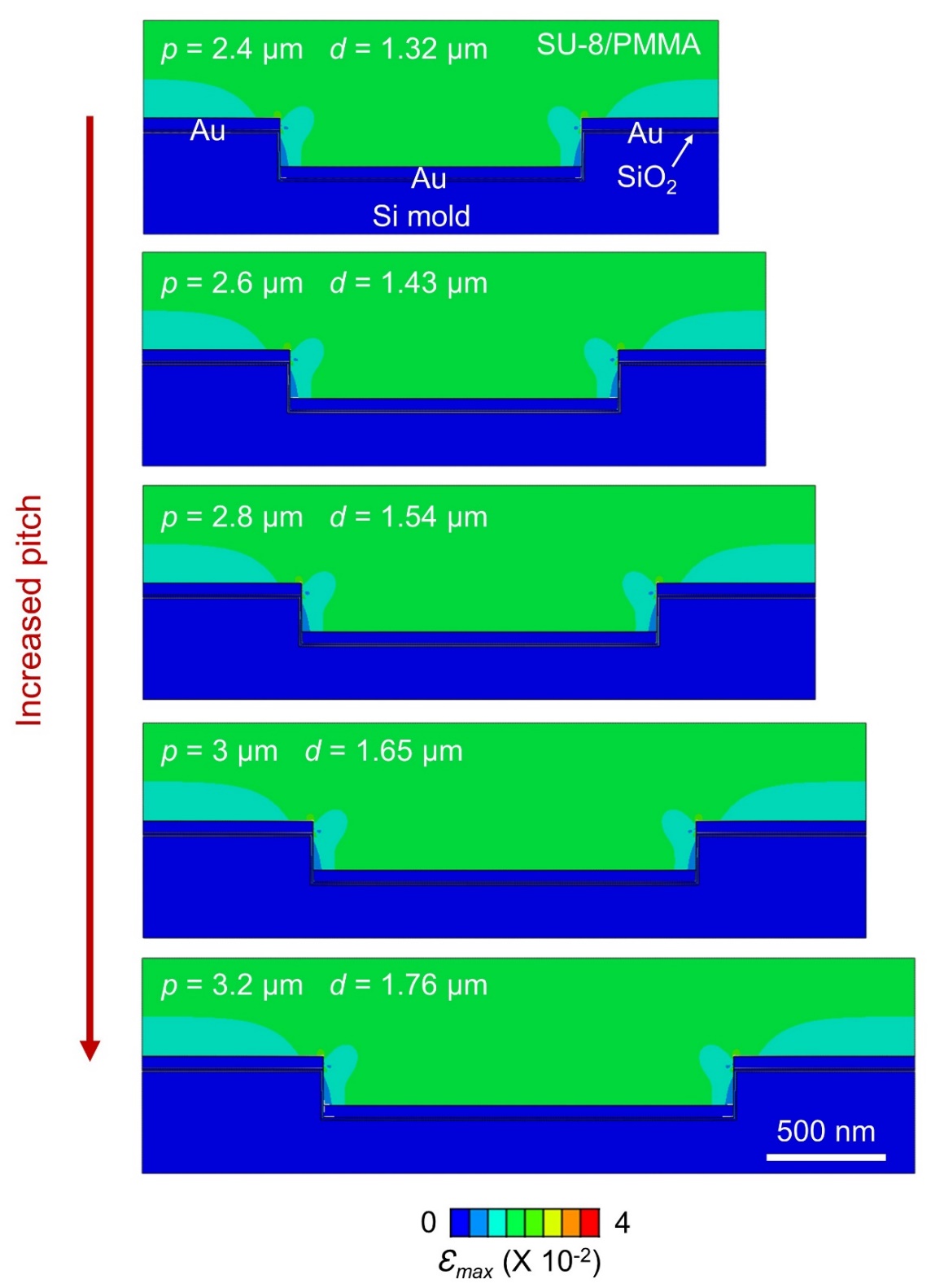


**Figure S4.** FEA results of strain distributions in the plasmonic film with different pitch sizes during the interfacial delamination.


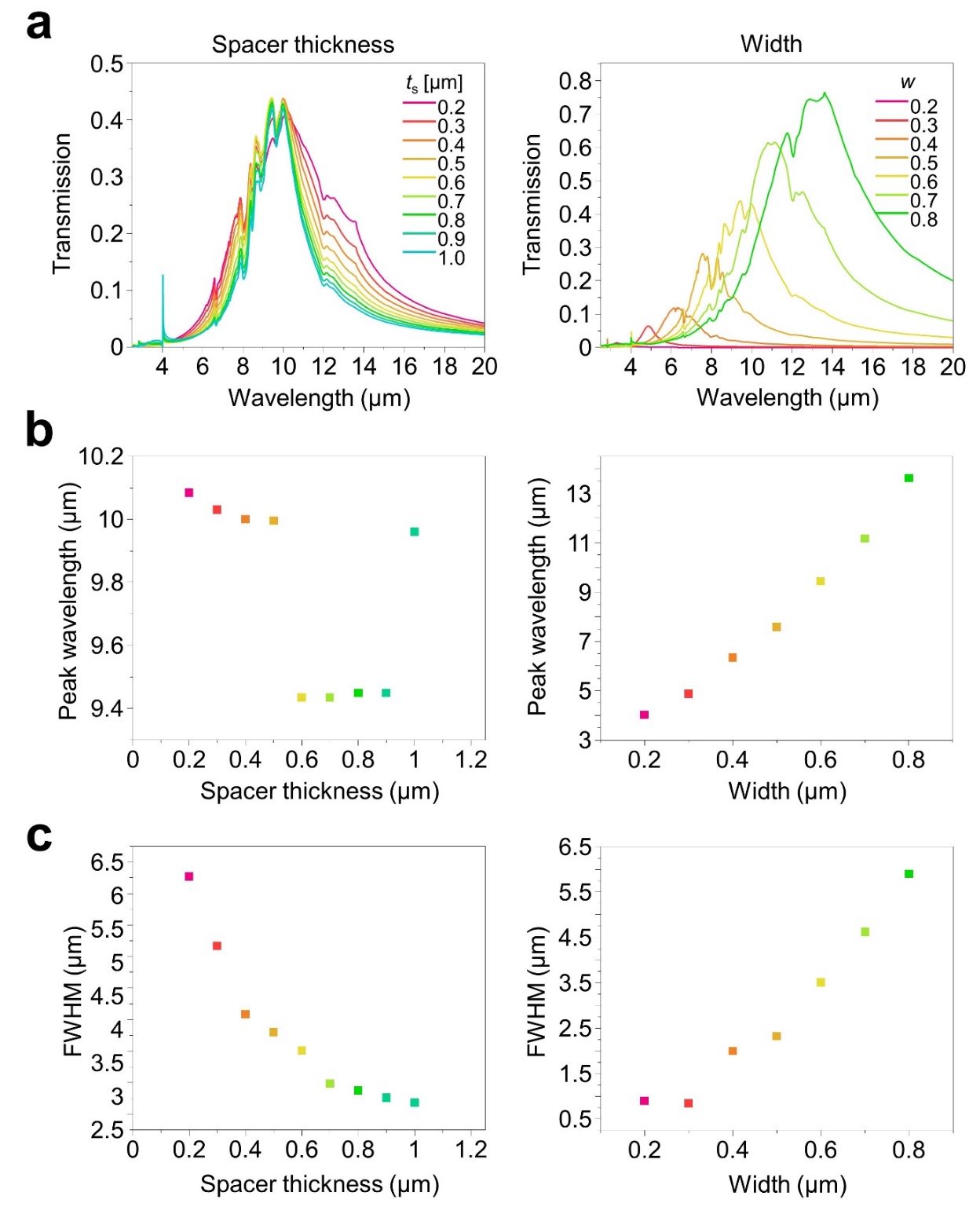


**Figure S5.** (a) Representative results of simulated transmission spectra for the quasi-3D nanoposts with variations in spacing (left) and width (right). (b) Peak wavelength and (c) FWHM (Full Width at Half Maximum) (bottom panel) for the quasi-3D nanoposts with variations in spacing (left) and width (right).


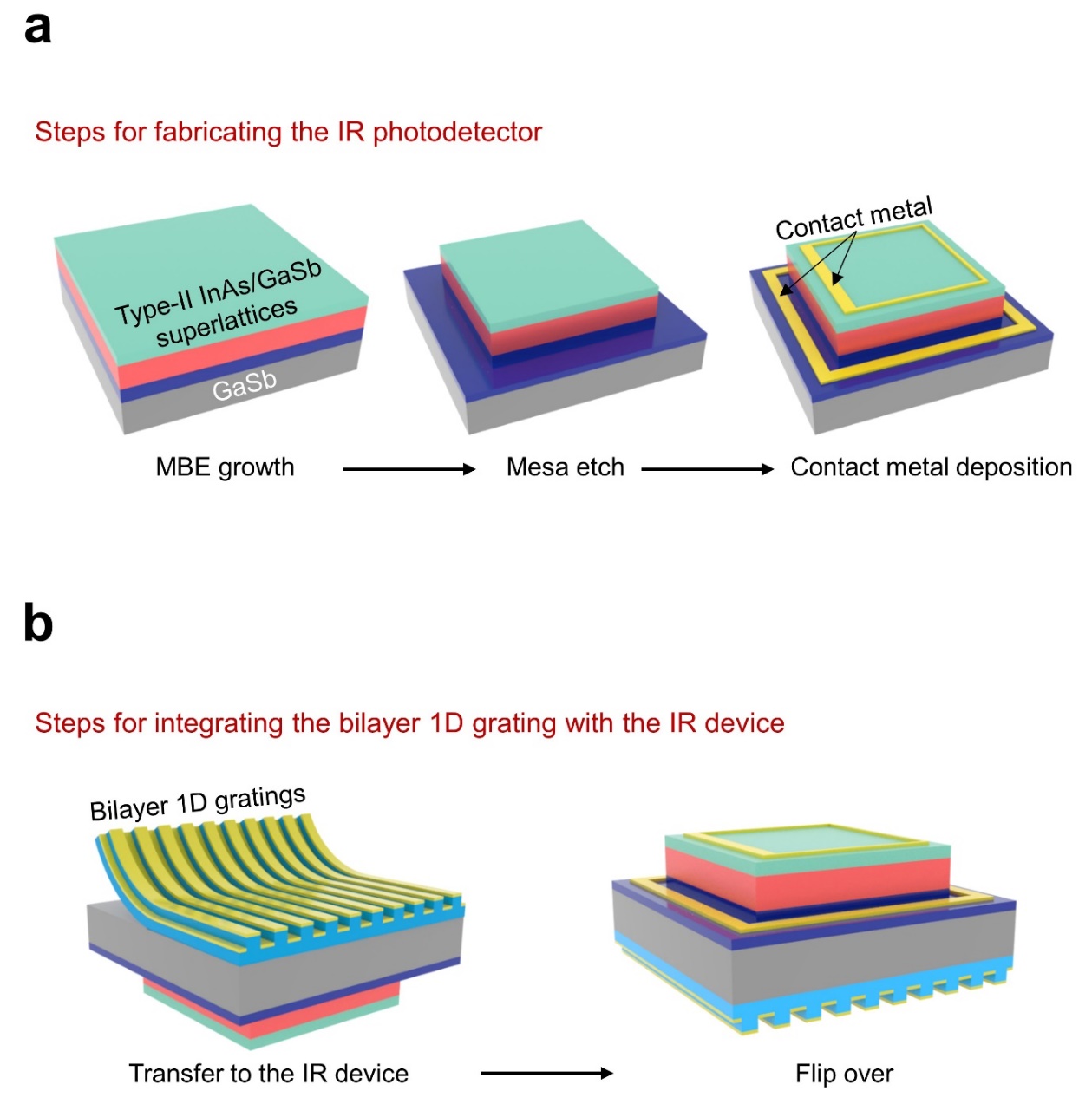


**Figure S6.** (a) Schematic illustrations describing the fabrication of the custom-designed long-wavelength infrared (LWIR) InAs/GaSb type-II superlattice (T2SL) based single pixel device (SPD). (b) Schematic illustrations of the fabrication procedures for monolithically integrating a polarization-sensitive custom plasmonic film with bilayer gratings onto the LWIR-T2SL SPD.


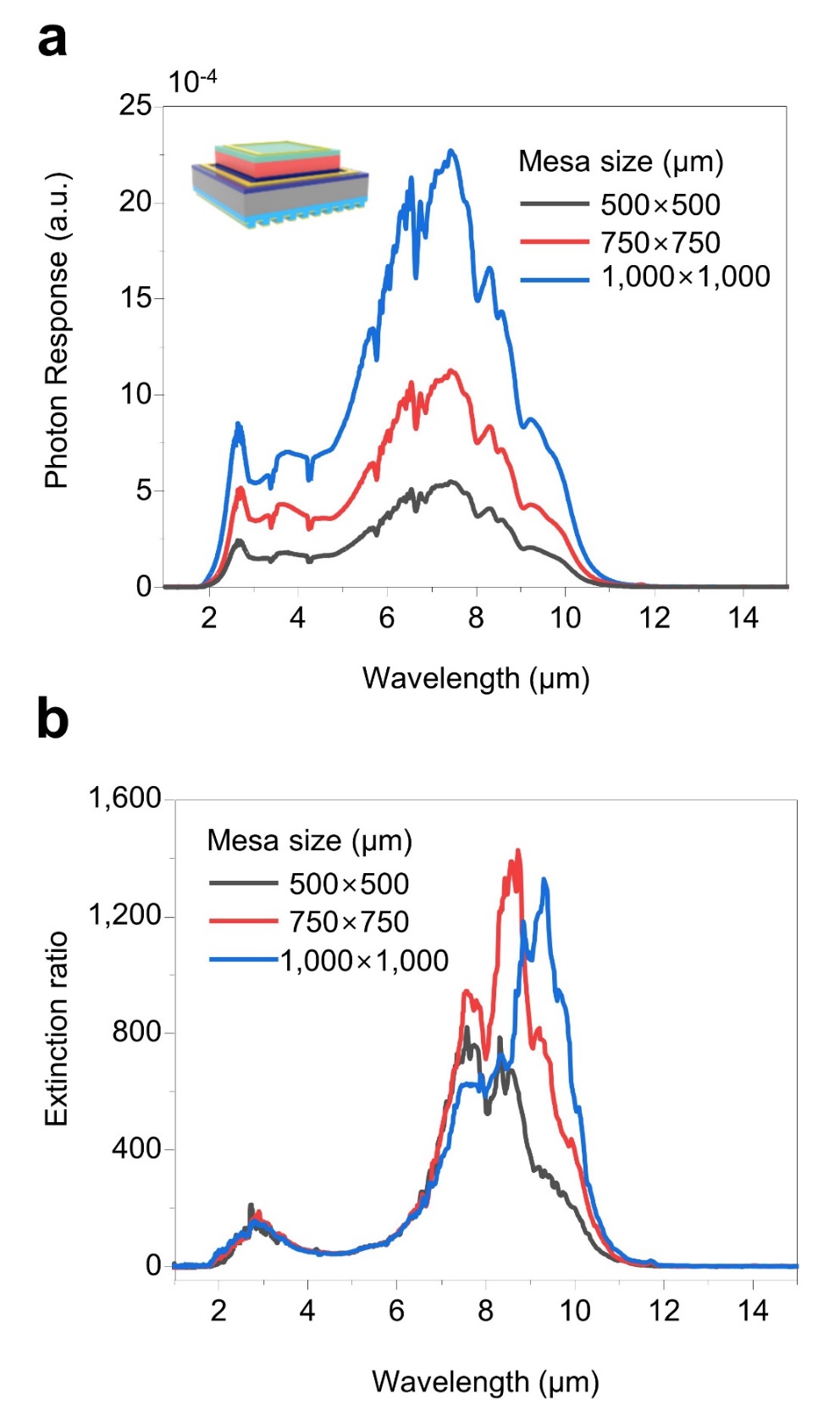


**Figure S7.** (a) Measured photon response and (b) extinction ratio of the monolithically integrated polarization-sensitive LWIR-T2SL photodetector for three mesa sizes: 500 × 500 μm², 750 × 750 μm², and 1,000 × 1,000 μm².

**Table S1.** Comparison of extinction ratio performances at the IR wavelength range for various reported infrared device types and utilized nanostructures.

| **Integrated polarization structure** | **Geometric parameters** | **Geometric parameters** | **Method** | **Wavelength [μm]** | **Extinction ratio** | **Ref.** |
| --- | --- | --- | --- | --- | --- | --- |
| 1D grating | *p* = 800 nm, *w* = 650 nm, *t*_Au_ = 50 nm | ZnO chip | Focused ion beam milling | 1.2 | 64 | 1 |
| 2D PdSe_2_ | - | Graphene/PdSe_2_/germanium heterojunction | - | 0.2 ~ 3.04 | 112.2 | 2 |
| 1D grating | *t*_Au_ = 200 nm | Quantum well infrared photodetectors | Photolithography | 13.1 ~ 13.7 | 136 | 3 |
| 1D grating | *p* = 300 nm, *w* = 150 nm, *t*_Au_ = 100 nm | InGaAs | Electron beam lithography | 1.064 | 21 | 4 |
| Metallic cavity | *p* = 400 nm | Quantum well infrared photodetectors | Semiconductor fabrication | 9.13 | 146 | 5 |
| bP/MoS_2_ | - | Black phosphorous (bP)/MoS_2_ heterojunction | - | 3.5 | 100 | 6 |
| Nanorods | *w* = 28.5 ~ 37.5nm | Lanthanide-doped upconversion nanoparticles | - | 1.55 | 6.14 | 7 |
| Nanostrip antennas | *p* = 5,000 nm, *t*_Au_ = 55 nm | Microbolometer | Electron beam lithography | 6.0~7.4 | 44.03 | 8 |
| - | *-* | - | Front-mounted | 6 | 353 | 9 |
| - | *-* | - | Front-mounted | 7.5–11.1 | 300 | 10 |
| **Bilayer 1D grating** | ***p* = 850 nm, *w* = 425 nm, *t*_Au_ = 100 nm** | **T2SL** | **Microbubble process** | **8.5** | **1,800** | **Our study** |

**Supplementary movie captions**

**Movie S1.** Separation process of the quasi-3D nanostructures via the microbubble process.

**Movie S2.** FEA results of the delamination process at the interface between the plasmonic film and the electrode substrate.

**Additional supplementary references**

[1] Y. Hou, H. Liang, A. Tang, X. Du, Z. Mei, *Applied Physics Letters* **2021**, 118, 063501.

[2] D. Wu, J. Guo, J. Du, C. Xia, L. Zeng, Y. Tian, Z. Shi, Y. Tian, X. J. Li, Y. H. Tsang, J. Jie, *ACS Nano* **2019**, 13, 9907.

[3] Y. W. Zhou, Z. F. Li, J. Zhou, N. Li, X. H. Zhou, P. P. Chen, Y. L. Zheng, X. S. Chen, W. Lu, *Scientific Reports* **2018**, 8, 15070.

[4] D. Sun, B. Feng, B. Yang, T. Li, X. Shao, X. Li, Y. Chen, *Opt. Lett.* **2020**, 45, 1559.

[5] X. Nie, H. Zhen, G. Huang, Y. Yin, S. Li, P. Chen, X. Zhou, Y. Mei, W. Lu, *Applied Physics Letters* **2020**, 116, 161107.

[6] J. Bullock, M. Amani, J. Cho, Y.-Z. Chen, G. H. Ahn, V. Adinolfi, V. R. Shrestha, Y. Gao, K. B. Crozier, Y.-L. Chueh, A. Javey, *Nature Photonics* **2018**, 12, 601.

[7] Y. Ji, G. Fang, J. Shang, X. Dong, J. Wu, X. Lin, W. Xu, B. Dong, *ACS Applied Materials & Interfaces* **2022**, 14, 50045.

[8] S. Jiang, J. Li, J. Li, J. Lai, F. Yi, *Opt. Express* **2022**, 30, 9065.

[9] S. Li, W. Jin, R. Xia, L. Li, X. Wang, *Opt. Express* **2016**, 24, 26414-26430.

[10] K. P. Gurton, S. McIntosh, *AD Report* **2014**
